# Alcohol Consumption Is a Risk Factor for Lower Extremity Arterial Disease in Chinese Patients with T2DM

**DOI:** 10.1155/2017/8756978

**Published:** 2017-07-06

**Authors:** Shanshan Yang, Shuang Wang, Bo Yang, Jinliang Zheng, Yuping Cai, Zhengguo Yang

**Affiliations:** ^1^Institute of Geriatrics, Beijing Key Laboratory of Ageing and Geriatrics, State Key Laboratory of Kidney Disease, Chinese PLA General Hospital, 28 Fuxing Road, Beijing 100853, China; ^2^Jinan Military Area CDC, Jinan, Shandong 250014, China; ^3^Department of Nephrology and Endocrinology, PLA 148th Hospital, Zibo 255300, China

## Abstract

**Objective:**

To investigate the relationship between alcohol consumption and diabetic lower extremity arterial disease (LEAD) in hospitalized patients with type 2 diabetes mellitus (T2DM).

**Methods:**

We evaluated 138 hospitalized patients with T2DM who consumed alcohol and 833 who did not. We used propensity score matching to reduce the confounding bias between groups. Additionally, a logistic regression analysis was performed with the matched data to evaluate the LEAD risk.

**Results:**

In total, 119 pairs of patients who did and did not consume alcohol were matched. According to the logistic regression analysis, patients who consumed >8 U of alcohol/day had a higher risk of LEAD (odds ratio (OR): 6.35, 95% confidence interval (CI): 1.78–22.65) than patients who did not consume alcohol. Additionally, after adjusting for age, gender, region, occupation, smoking status, body mass index, weight change, and duration of diabetes, the OR of peripheral artery disease after >20 years of alcohol consumption was 3.48 (95% CI: 1.09–11.15). Furthermore, we observed a significant dose-response relationship between alcohol consumption and LEAD.

**Conclusions:**

Alcohol consumption may be a risk factor of LEAD in patients with T2DM. Patients with T2DM should be advised to stop drinking, to prevent the onset of LEAD.

## 1. Introduction

Lower extremity arterial disease (LEAD) is one of the most common complications of diabetes and harms the peripheral arteries via multiple pathways [1]. LEAD is also associated with healing failure, amputation, cardiovascular events, and an increased risk of premature mortality [2, 3]. Furthermore, LEAD is a pathological process, and only one in four LEAD patients survives for more than 10 years. Approximately 10 million men and women in the United States suffer from LEAD [4], and in Chinese patients with diabetes older than 50 years, the prevalence of LEAD ranges from 16.9 to 23.8% [5]. Compared with individuals without diabetes mellitus (DM), patients with DM have a 2-fold higher risk of LEAD and of having an earlier onset age, a more serious illness, more lesions, and a worse prognosis [6]. The rate at which patients with LEAD eventually require an amputation is as high as 33% [7]. In 1997, the medical cost of a diabetic foot ulcer in the United States was $10,831 and the average hospital stay length was 8.9 days; the medical cost of an amputation was $17,302 and the hospital stay length was 12 days, far exceeding the lengths of stay for coronary bypass surgery (9.9 days) and myocardial infarction (6.9 days) [8]. In China, the average duration of hospitalization for patients with LEAD undergoing amputation was 26 days and the average cost was 14,906 yuan [5].

Considering the serious health hazards of LEAD, a comprehensive identification of preventable risk factors of LEAD is important to improving patients' quality of life and reducing the associated medical costs. Smoking, ageing, race/ethnicity, increased levels of inflammatory markers, homocysteinaemia, and abdominal obesity are currently identified as risk factors for LEAD [9]. However, the association between alcohol use and LEAD remains unclear. Alcohol consumers with peripheral artery disease (PAD) were reported to have a lower mortality than patients with PAD who did not consume alcohol. On the other hand, heavy drinking has been reported to be a risk factor for PAD, whereas other studies have not identified an association between alcohol use and LEAD [9–12]. In addition, in these studies, the measures of alcohol consumption were not consistent, and the demographic characteristics of the groups who did and did not consume alcohol differed significantly. Studies on this topic in the Chinese population are limited. Thus, we designed a study to assess the association between alcohol consumption and LEAD in Chinese patients with type 2 diabetes mellitus (T2DM) using U (an international measure of ethanol) to measure alcohol consumption and propensity score matching (PSM) to control for differences in characteristics between patients who did and did not consume alcohol.

## 2. Design and Methods

### 2.1. Study Sample

We used clinical data from the Department of Nephrology and Endocrinology, PLA 148th Hospital. Of the 1025 inpatients (from January 2010 to December 2012), we excluded 25 inpatients with type 1 DM and 11 adult inpatients with latent autoimmune diabetes, and we recruited 989 (507 men and 482 women) as our participants.

We collected data regarding each participant's gender, age, occupation, region of residence, alcohol and smoking habits, T2DM duration, and LEAD status.

### 2.2. Measurements

T2DM was defined according to the American Diabetes Association criteria [13]. LEAD was diagnosed as follows: (1) an ankle-brachial index (ABI) < 0.90, which is the systolic blood pressure at the ankle divided by the systolic blood pressure of the arm; (2) an ABI > 1.3 and a toe-brachial index (TBI) < 0.7; and (3) intermittent claudication, 0.9 < ABI < 1.3, and an ABI that is reduced by 15–20% after an exercise tolerance test [14]. The ABI was measured in all subjects, and the TBI was measured in subjects with an ABI > 1.3 using a Vista AVS system (Summit Doppler Systems Inc., Golden, CO 80403, USA).

An alcohol user was defined as a regular drinker who consumed alcohol almost every day and had regularly consumed alcohol for more than half a year. This definition was used because the average alcohol consumption of occasional drinkers is difficult to determine [15]. The participants' alcohol use status was defined by asking the question “Are you a regular drinker who has used alcohol almost every day for more than a half year?” The duration and quantity of alcohol consumption were defined by asking the questions “How many years have you been a regular drinker?” and “Do you drink more than two standard glasses (approximately 250 ml) of white spirits or 2.5 bottles/5 cans (approximately 1,500 ml) of beer a day on average?” The answers to these questions were either “A. Yes” or “B. No.” A volume of 1 U of consumed alcohol equals 10 ml or 8 g of ethanol, which corresponds to the amount of alcohol a normal (60 kg) adult can metabolize in 1 hour [16]. Thus, we defined a heavy drinker as someone who consumed 8 U of alcohol a day on average, that is, a person who metabolized alcohol at least 8 hours every day.

This information was collected by a primary nurse. The patients' answers to the questions on alcohol use were confirmed by the patients and their relatives to ensure the accuracy of the information. Central obesity was defined as a waist circumference (WC) > 90 cm in men and >80 cm in women [17].

### 2.3. Statistical Analysis

SPSS version 19.0 was used to analyse the data. The significance level for all tests was set at a two-tailed *α* value of 0.05. The differences in means and proportions were evaluated using Student's *t*-test and the chi-square test, respectively. Logistic regression models were used to identify the risk of alcohol use.

PSM [18] was used to match the groups of those who did and did not consume alcohol. Gender, age, region, occupation, smoking status, body mass index (BMI), WC, and the duration of T2DM were included as covariates. We used the nearest-neighbour matching to match former smokers with current smokers at a 1 : 1 ratio with a calliper width of 0.02 [19].

### 2.4. Ethical Considerations

The Committee for Medical Ethics of the Chinese PLA General Hospital examined and approved our study. Before completing the questionnaire, each involved participant signed an informed consent form.

## 3. Results

Nine hundred and eighty-nine (507 men and 482 women) inpatients were involved in our study before PSM. The average age was 56.8 ± 11.6 years (range: 14–93 years). The average ages of patients who did and did not consume alcohol were 52.3 ± 11.3 years (range: 28–85 years) and 57.5 ± 11.3 years (range: 14–93 years), respectively. The general characteristics (age, gender, origin, occupation, smoking status, BMI, and central obesity) of the participants are shown in Table 1. Compared with the group of alcohol consumers, the group of patients who did not consume alcohol comprised more women, more workers engaged in hard physical labour, fewer smokers, and patients who were older and had more central obesity and longer durations of T2DM (5.4 ± 6.0 years versus 7.5 ± 6.6 years; *P* < 0.05).

After PSM, 238 participant pairs were matched, and the two groups were balanced for age, gender, occupation, smoking status, BMI, central obesity, and T2DM duration (with and without alcohol consumption: 5.8 ± 6.3 years versus 5.9 ± 6.1 years, resp.; *P* = 0.937) (Table 1).

According to the logistic regression analysis, patients who consumed alcohol had a higher risk of LEAD (OR: 2.75, 95% CI: 1.11–6.80) than patients who did not after adjusting for age, gender, region, occupation, smoking status, BMI, WC, and T2DM duration. Regarding the risk of LEAD after adjusting for alcohol use ≤8 U/day and >8 U/day, the odds ratios (ORs) were 2.07 (95% confidence interval (CI): 0.78–5.54, *P* > 0.05) and 6.35 (95% CI: 1.78–22.65, *P* < 0.05), respectively. We also observed a dose-response relationship between the units of alcohol consumed per day and LEAD risk (after adjusting for age, gender, region, occupation, smoking status, BMI, WC, and T2DM duration, *P* = 0.005). In addition, compared with patients who did not consume alcohol, patients who had consumed alcohol for >20 years had a higher risk of LEAD after adjusting for various factors (OR: 3.48, 95% CI: 1.09–11.15), and a dose-response relationship between the number of years of alcohol use and the risk of LEAD was also observed (*P* = 0.019) (Table 2).

When alcohol consumption was analysed as a continuous outcome, models A to C showed that increased alcohol consumption was associated with an increased risk of LEAD (all *P* < 0.05, Table 2). However, the association between continuous years of alcohol consumption and LEAD was not significant after adjusting for various factors (Table 2). Furthermore, we utilized model D (adjusting for age, gender, region, occupation, smoking status, BMI, WC, T2DM duration, systolic blood pressure, cholesterol, and prevalent cardiovascular disease) to analyse participants with cholesterol, blood pressure, and prevalent cardiovascular disease data (*n* = 185) and obtained similar results (Table S1 available online at https://doi.org/10.1155/2017/8756978).

The gender imbalance between alcohol consumers and nonconsumers is striking. We performed an analysis on male patients only (the number of alcohol-consuming women was too low for a separate analysis of female patients). Compared with male patients who did not consume alcohol, male patients who consumed alcohol had a higher risk of LEAD (OR: 3.17, 95% CI: 1.25–8.09) after adjusting for age, region, occupation, smoking status, BMI, WC, and T2DM duration. Regarding the risk of LEAD after adjusting for alcohol use ≤8 U/day and >8 U/day, the ORs were 2.43 (95% CI: 0.89–6.68, *P* > 0.05) and 7.03 (95% CI: 1.91–25.84, *P* < 0.05), respectively. We also observed a dose-response relationship between the units of alcohol consumed per day and LEAD risk (after adjusting for age, region, occupation, smoking status, BMI, WC, and T2DM duration, *P* = 0.003). In addition, compared with male patients who did not consume alcohol, male patients who had consumed alcohol for >20 years had a higher risk of LEAD after adjusting for various factors (OR: 2.82, 95% CI: 1.07–7.91), and a dose-response relationship between the number of years of alcohol use and the risk of LEAD was also observed (*P* = 0.039) (Table 3).

When alcohol consumption was analysed as a continuous outcome, models A to C showed that increased alcohol consumption was associated with an increased risk of LEAD (all *P* < 0.05, Table 3). In addition, the association between continuous years of alcohol consumption and LEAD was not significant after adjusting for various factors (Table 3). Furthermore, we utilized model D (adjusting for age, origin, occupation, smoking status, BMI, WC, T2DM duration, systolic blood pressure, cholesterol, and prevalent cardiovascular disease) to analyse male participants with cholesterol, blood pressure, and prevalent cardiovascular disease data (*n* = 182) and obtained similar results (Table S2).

## 4. Discussion

In this study, we observed a significant association between alcohol consumption and LEAD in patients with T2DM. We used a standard and universal measure of alcohol consumption; in China, individuals usually drink white spirits distilled from sorghum or maize or beer, and “liang” (50 g) is usually used as the measurement for the amount of alcohol consumed [20]. These differences from Western countries make comparisons between nations difficult; however, we used the standard and universal measure U, that is, 10 ml or 8 g of ethanol, to solve this problem. We also used PSM to comprehensively control and adjust for a wide range of potential confounders and to improve the comparability between the two groups.

As shown in a study by Mukamal et al. [10], older adults in Pennsylvania who consumed 1–13 drinks per week had a lower risk (OR: 0.56, 95% CI: 0.33–0.95) of hospitalization for LEAD; however, this reduced risk became insignificant when >13 drinks were consumed per week. This study used drinks per week as a measurement of alcohol consumption and did not present information about the quantity of alcohol consumed or the years of alcohol consumption. Vliegenthart et al. [11] observed an inverse relationship between alcohol consumption and PAD in nonsmoking women, but not in nonsmoking men; this difference may be related to the propensity of males to drink beer, wine, and liquor, whereas females predominantly drink wine and types of fortified wine. In addition, Xie et al. [21] observed an association between heavy drinking (>60 g/day) and a higher risk (OR: 2.878, 95% CI: 1.215–4.018) of a low ABI in Chinese men; this result is consistent with the findings of our study. Furthermore, heavy drinking has adverse effects on blood pressure and serum triglyceride levels [22], both of which may lead to LEAD.

This study had several limitations. As the information on alcohol consumption was based on recall, recall bias could not be completely excluded; however, the information was confirmed by patients and their relatives to ensure accuracy. Second, our sample may not be completely representative of patients with T2DM in China because our hospital is one of the best hospitals in Zibo, and the inpatients here have higher proportions of diabetic complications. However, the representativeness of our sample should not substantially affect the internal validity of this study. Third, the cholesterol, blood pressure, and prevalent cardiovascular disease data were missing for 229 participants, and thus, we did not include these three variables as confounders for PSM. Furthermore, we did not collect information about physical activity or homocysteine levels, which are also risk factors for LEAD [23]. However, we utilized model D (adjusting for age, gender, region, occupation, smoking status, BMI, WC, T2DM duration, systolic blood pressure, cholesterol, and prevalent cardiovascular disease) in participants with available cholesterol, blood pressure, and prevalent cardiovascular disease data (*n* = 185) and obtained similar results, as the effect of alcohol consumption on the LEAD risk was still significant after adjusting for these factors. Finally, we could not examine the hazard ratio of alcohol consumption with respect to LEAD because detailed information regarding the onset time of LEAD was not available.

In summary, our study observed a dose-response relationship between alcohol consumption and LEAD among inpatients with T2DM. We used a standard and universal measurement of alcohol consumption and increased the comparability of the two groups using the PSM method. Alcohol consumption may be a risk factor for LEAD in patients with T2DM; however, further cohort studies should be conducted to verify the causal relationship. Based on our findings, patients with T2DM should be advised to stop drinking, or at least to avoid heavy drinking, to prevent the onset of LEAD.

## Supplementary Material

Table S1 OR (95% CI) of LEAD in participants according to alcohol use (n=185). Table S2 OR (95% CI) of LEAD in male participants according to alcohol use (n=182).

## Figures and Tables

**Table 1:** Demographic characteristics of the participants according to alcohol use before and after PSM.

Group	Number (%)	Alcohol use (before PSM)			Alcohol use (after PSM)		
	Total *n* = 971	Yes (*n* = 138)	No (*n* = 833)	*P*	Yes (*n* = 119)	No (*n* = 119)	*P*
Age (years)				<0.001			0.534
≤40	81 (8.3)	22 (15.9)	59 (7.1)		16 (13.4)	15 (12.6)	
60–69	529 (54.5)	79 (57.2)	450 (54.0)		68 (57.1)	76 (63.9)	
≥70	361 (37.2)	37 (26.8)	324 (38.9)		35 (29.4)	28 (23.5)	
Gender				<0.001			1.000
Male	498 (51.3)	136 (98.6)	362 (43.5)		117 (98.3)	117 (98.3)	
Female	473 (48.7)	2 (1.4)	471 (56.5)		2 (1.7)	2 (1.7)	
Occupation				0.01			0.562
White collar	103 (10.6)	22 (15.9)	81 (9.7)		84 (70.6)	77 (64.7)	
Light physical labourer	117 (12.0)	23 (16.7)	94 (11.3)		19 (16.0)	25 (21.0)	
Hard physical labourer	751 (77.3)	93 (67.4)	658 (79.0)		16 (13.4)	17 (14.3)	
Region				0.756			0.408
Shandong province	940 (96.8)	133 (96.4)	807 (96.9)		117 (98.3)	115 (96.6)	
Other province	31 (3.2)	5 (3.6)	26 (3.1)		2 (1.7)	4 (3.4)	
Smoker				<0.001			0.512
Yes	182 (18.7)	90 (65.2)	92 (11.0)		71 (59.7)	66 (55.5)	
No	789 (81.3)	48 (34.8)	741 (89.0)		48 (40.3)	53 (44.5)	
BMI				0.038			0.783
<24.00	368 (37.9)	39 (28.3)	329 (39.5)		36 (30.3)	32 (26.9)	
24.00–27.99	388 (40.0)	62 (44.9)	326 (39.1)		50 (42.0)	55 (46.2)	
≥28.00	215 (22.1)	37 (26.8)	178 (21.4)		33 (27.7)	32 (26.9)	
Central obesity				<0.001			0.697
Yes	625 (64.4)	66 (47.8)	559 (67.1)		60 (50.4)	57 (47.9)	
No	346 (35.6)	72 (52.2)	274 (32.9)		59 (49.6)	62 (52.1)	
Mean ± SD							
Age		52.3 ± 11.3	57.5 ± 11.3	<0.001	53.2 ± 11.9	51.9 ± 11.9	0.401
Duration of T2DM		5.4 ± 6.0	7.5 ± 6.6	0.001	5.8 ± 6.3	5.9 ± 6.1	0.937
BMI		25.8 ± 3.7	25.3 ± 4.1	0.143	25.8 ± 3.9	25.9 ± 3.9	0.861
WC		90.9 ± 8.4	88.3 ± 8.8	0.001	90.9 ± 8.7	90.7 ± 8.4	0.825

**Table 2:** OR (95% CI) of LEAD in participants according to alcohol use.

	*n* (%)	Model A	Model B	Model C
		OR (95% CI)	OR (95% CI)	OR (95% CI)
Alcohol use				
No (reference)	9 (7.6)	1	1	1
Yes	21 (17.6)	2.62 (1.15–5.99)	2.61 (1.09–6.23)	2.75 (1.11–6.80)
*P*		0.022	0.031	0.028
Alcohol consumption				
No (reference)	9 (7.6)	1	1	1
≤8 U/day	14 (15.7)	2.28 (0.94–5.54)	1.95 (0.76–5.02)	2.07 (0.78–5.54)
>8 U/day	7 (23.3)	3.72 (1.26–11.01)	6.33 (1.89–21.15)	6.35 (1.78–22.65)
*P* for trend		0.012	0.004	0.005
Alcohol use duration				
No (reference)	9 (7.6)	1	1	1
≤20 years	8 (15.1)	2.17 (0.79–5.99)	2.25 (0.86–5.90)	2.41 (0.88–6.60)
>20 years	13 (19.7)	3.00 (1.21–7.45)	3.40 (1.13–10.23)	3.48 (1.09–11.15)
*P* for trend		0.017	0.015	0.019
Continuous				
No (reference)		1	1	1
Alcohol consumption (U)		1.06 (1.00–1.12)	1.10 (1.03–1.18)	1.11 (1.04–1.19)
*P*		0.048	0.004	0.003
No (reference)		1	1	1
Alcohol use duration (years)		1.03 (1.01–1.06)	1.02 (0.99–1.04)	1.02 (0.99–1.05)
*P*		0.013	0.054	0.055

Model A: crude model; model B: adjusted for age, gender, region, and occupation; model C: adjusted for age, gender, region, occupation, smoking status, BMI, WC, and T2DM duration.

**Table 3:** OR (95% CI) of LEAD in male participants according to alcohol use.

	*n* (%)	Model A	Model B	Model C
		OR (95% CI)	OR (95% CI)	OR (95% CI)
Alcohol use				
None (reference)	8 (6.8)	1	1	1
Yes	21 (17.9)	2.98 (1.26–7.04)	3.10 (1.25–7.67)	3.17 (1.25–8.09)
*P*		0.013	0.015	0.016
Alcohol consumption				
None (reference)	8 (6.8)	1	1	1
≤8 U/day	14 (16.1)	2.61 (1.04–6.54)	2.36 (0.88–6.26)	2.43 (0.89–6.68)
>8 U/day	7 (23.3)	4.15 (1.37–12.58)	7.32 (2.10–25.42)	7.03 (1.91–25.84)
*P* for trend		0.007	0.002	0.003
Alcohol use duration				
None (reference)	8 (6.8)	1	1	1
≤20 years	8 (15.1)	2.42 (0.86–6.85)	3.88 (1.25–12.07)	3.93 (1.19–12.96)
>20 years	13 (20.3)	3.47 (1.36–8.90)	2.74 (1.01–7.39)	2.82 (1.01–7.91)
*P* for trend		0.009	0.042	0.039
Continuous				
None (reference)		1	1	1
Alcohol consumption (U)		1.06 (1.00–1.13)	1.11 (1.04–1.18)	1.11 (1.04–1.19)
*P*		0.036	0.003	0.003
None (reference)		1	1	1
Alcohol use duration (years)		1.04 (1.01–1.07)	1.03 (1.00–1.05)	1.03 (1.00–1.06)
*P*		0.004	0.067	0.063

Model A: crude model; model B: adjusted for age, region, and occupation; model C: adjusted for age, region, occupation, smoking status, BMI, WC, and T2DM duration.

## Abbreviations

ABI: Ankle-brachial index
BMI: Body mass index
95% CI: 95% confidence interval
DM: Diabetes mellitus
LEAD: Lower extremity arterial disease
OR: Odds ratio
PAD: Peripheral artery disease
PSM: Propensity score matching
T2DM: Type 2 diabetes mellitus
TBI: Toe-brachial index
WC: Waist circumference.
