# Supplementary material for: Alcohol Consumption Is a Risk Factor for Lower Extremity Arterial Disease in Chinese Patients with T2DM
Source: J Diabetes Res. 2017 Jul 6;2017:8756978. doi: 10.1155/2017/8756978 (PMC5518525; doi:10.1155/2017/8756978)
Supplement: Supplementary file 1 — Table S1 OR (95% CI) of LEAD in participants according to alcohol use (n=185). Table S2 OR (95% CI) of LEAD in male participants according to alcohol use (n=182). [file 8756978.f1.pdf]

Table S1 OR (95% CI) of LEAD in participants according to alcohol use (n=185)

		N (%)	Model D<br>OR (95% CI)
Alcohol use			
	None (reference)	8 (9.2)	1
	Yes	19 (19.4)	**3.14 (1.07-9.17)**
	P		0.037
Alcohol consumption			
	None (reference)	8 (9.2)	1
	≤8U/day	12 (16.7)	2.22 (0.69-7.17)
	>8U/day	7 (26.9)	**7.15 (1.70-30.10)**
	P for trend		0.009
Alcohol use duration			
	None (reference)	8 (9.2)	1
	≤20years	7 (15.6)	3.73 (0.96-14.53)
	>20years	12 (22.6)	2.83 (0.86-9.27)
	P for trend		0.074
Continuous			
	None (reference)		1
	Alcohol consumption (U)		**1.12 (1.03-1.20)**
	P		0.005
	None (reference)		1
	Alcohol use duration (years)		1.02 (0.99-1.05)
	P		0.191

Model D: Adjusted for age, gender, region, occupation, smoking status, BMI, WC, T2DM duration, systolic blood pressure, cholesterol and prevalent cardiovascular disease.

Table S2 OR (95% CI) of LEAD in male participants according to alcohol use (n=182)

		N (%)	Model D<br>OR (95% CI)
Alcohol use			
	None (reference)	7 (8.1)	1
	Yes	19 (19.8)	**3.52 (1.17-10.60)**
	P		0.025
Alcohol consumption			
	None (reference)	7 (8.1)	1
	≤8U/day	12 (17.1)	2.52 (0.76-8.37)
	>8U/day	7 (26.9)	**7.77 (1.80-33.54)**
	P for trend		0.006
Alcohol use duration			
	None (reference)	7 (8.1)	1
	≤20years	7 (15.6)	**3.92 (1.00-15.35)**
	>20years	12 (23.5)	3.30 (0.98-11.12)
	P for trend		0.047
Continuous			
	None (reference)		1
	Alcohol consumption (U)		**1.12 (1.03-1.20)**
	P		0.004
	None (reference)		1
	Alcohol use duration (years)		1.03 (1.00-1.06)
	P		0.089

Model D: Adjusted for age, region, occupation, smoking status, BMI, WC, T2DM duration, systolic blood pressure, cholesterol and prevalent cardiovascular disease.
