# Supplementary material for: Alcohol Consumption Is a Risk Factor for Lower Extremity Arterial Disease in Chinese Patients with T2DM
Source: J Diabetes Res. 2017 Jul 6;2017:8756978. doi: 10.1155/2017/8756978 (PMC5518525; doi:10.1155/2017/8756978)
Supplement: Supplementary file 2 [file 8756978.f2.doc]

| **Table S1 OR (95% CI) of LEAD in participants according to alcohol use (n=185)** | | | | |
| --- | --- | --- | --- | --- |
|  |  | | N (%) | Model D |
|  |  |  |  | OR (95% CI) |
| Alcohol use | | |  |  |
|  | | None (reference) | 8 (9.2) | 1 |
|  | | Yes | 19 (19.4) | **3.14 (1.07-9.17)** |
|  | | P |  | 0.037 |
| Alcohol consumption | |  |  |  |
|  | | None (reference) | 8 (9.2) | 1 |
|  | | ≤8U/day | 12 (16.7) | 2.22 (0.69-7.17) |
|  | | >8U/day | 7 (26.9) | **7.15 (1.70-30.10)** |
|  | | P for trend |  | 0.009 |
| Alcohol use duration | |  |  |  |
|  | | None (reference) | 8 (9.2) | 1 |
|  | | ≤20years | 7 (15.6) | 3.73 (0.96-14.53) |
|  | | >20years | 12 (22.6) | 2.83 (0.86-9.27) |
|  | | P for trend |  | 0.074 |
| Continuous | |  |  |  |
|  | | None (reference) |  | 1 |
|  | | Alcohol consumption (U) |  | **1.12 (1.03-1.20)** |
|  | | P |  | 0.005 |
|  | |  |  |  |
|  | | None (reference) |  | 1 |
|  | | Alcohol use duration (years) |  | 1.02 (0.99-1.05) |
|  | | P |  | 0.191 |

Model D: Adjusted for age, gender, region, occupation, smoking status, BMI, WC, T2DM duration, systolic blood pressure, cholesterol and prevalent cardiovascular disease.

| **Table S2 OR (95% CI) of LEAD in male participants according to alcohol use (n=182)** | | | |
| --- | --- | --- | --- |
|  |  | N (%) | Model D |
|  |  |  | OR (95% CI) |
| Alcohol use | |  |  |
|  | None (reference) | 7 (8.1) | 1 |
|  | Yes | 19 (19.8) | **3.52 (1.17-10.60)** |
|  | P |  | 0.025 |
| Alcohol consumption |  |  |  |
|  | None (reference) | 7 (8.1) | 1 |
|  | ≤8U/day | 12 (17.1) | 2.52 (0.76-8.37) |
|  | >8U/day | 7 (26.9) | **7.77 (1.80-33.54)** |
|  | P for trend |  | 0.006 |
| Alcohol use duration |  |  |  |
|  | None (reference) | 7 (8.1) | 1 |
|  | ≤20years | 7 (15.6) | **3.92 (1.00-15.35)** |
|  | >20years | 12 (23.5) | 3.30 (0.98-11.12) |
|  | P for trend |  | 0.047 |
| Continuous |  |  |  |
|  | None (reference) |  | 1 |
|  | Alcohol consumption (U) |  | **1.12 (1.03-1.20)** |
|  | P |  | 0.004 |
|  |  |  |  |
|  | None (reference) |  | 1 |
|  | Alcohol use duration (years) |  | 1.03 (1.00-1.06) |
|  | P |  | 0.089 |

Model D: Adjusted for age, region, occupation, smoking status, BMI, WC, T2DM duration, systolic blood pressure, cholesterol and prevalent cardiovascular disease.
